# Computed tomography dose management practices and the adoption of automated dose monitoring tools in Australia: a national survey

**DOI:** 10.1093/rpd/ncaf109

**Published:** 2025-09-23

**Authors:** Mohammed Alanazi, Peter Kench, Seyedamir Tavakoli-Taba, Ernest Ekpo

**Affiliations:** Medical Image Optimisation and Perceptions Group, Discipline of Medical Imaging Science, Faculty of Medicine and Health, Susan Wakil Health Building, Camperdown Campus, Western Avnue,The University of Sydney, NSW 2006, Australia; Department of Radiology Sciences, Faculty of Applied Medical Sciences, Majmaah University, Al Majmaah 11952, Saudi Arabia; Medical Image Optimisation and Perceptions Group, Discipline of Medical Imaging Science, Faculty of Medicine and Health, Susan Wakil Health Building, Camperdown Campus, Western Avnue, The University of Sydney, NSW 2006, Australia; Medical Image Optimisation and Perceptions Group, Discipline of Medical Imaging Science, Faculty of Medicine and Health, Susan Wakil Health Building, Camperdown Campus, Western Avnue,The University of Sydney, NSW 2006, Australia; Medical Image Optimisation and Perceptions Group, Discipline of Medical Imaging Science, Faculty of Medicine and Health, Susan Wakil Health Building, Camperdown Campus, Western Avnue,The University of Sydney, NSW 2006, Australia

## Abstract

This study investigates computed tomography (CT) dose management and automatic dose monitoring software (DMS) use in Australian radiology departments. An online survey covering departmental characteristics, dose management practices, DMS usage, and radiation protection activities was distributed through national radiation organisations and social media. Of the 132 initial responses received, 45 were eligible, completed, and analysed. The findings indicate 91% (*n* = 41/45) of departments regularly assess CT doses, with 63% (*n* = 26/41) conducting such assessments only once a year. Automatic DMS tools were utilised by 41% (*n* = 17/41) of these departments for CT dose data collection and analysis, while 59% (*n* = 24/41) relied on traditional methods. Only 25% (*n* = 6/24) of non-DMS users indicated plans for future adoption. A radiation protection team was present in 41% (*n* = 17/41) of departments. While dose assessment is commonly practised, it mostly relies on manual methods and is performed infrequently. Broader DMS implementation and formal radiation protection teams are recommended to strengthen CT dose management and enable continuous monitoring.

## Introduction

Computed tomography (CT) has become an indispensable tool in modern medicine, producing rapid and detailed cross-sectional images of the body that facilitate the detection, evaluation, and diagnosis of a wide array of clinical conditions, ranging from trauma to complex disease processes [1, 2]. The increasing reliance on CT imaging over the past decades has led to a parallel increase in attention to the radiation doses associated with these examinations [3]. Studies have consistently shown that CT delivers substantially higher doses of radiation compared to conventional radiographic procedures [4, 5]. Of particular concern are paediatric patients and individuals requiring multiple CT examinations, given the elevated lifetime risk of radiation-induced malignancies [6–8]. Various international professional organisations have issued guidelines to improve patient safety during ionising imaging procedures, including CT [9, 10]. These guidelines emphasise the justification of imaging requests, optimisation of scan protocols in line with the ‘As Low as Reasonably Practicable’ principle, and the application of diagnostic reference levels (DRLs) tailored to national or regional standards [9, 10]. In parallel, various dose-saving technologies have been introduced, including automatic tube potential selection, tube current modulation, and advanced image reconstruction methods such as iterative reconstruction and deep learning algorithms. These technologies tailor radiation exposure to each patient’s size and diagnostic task, while also controlling image noise, thereby minimising dose without compromising diagnostic image quality [11–13]. Additionally, ongoing monitoring and programmatic review of local radiation dose data are recommended to ensure imaging facilities maintain high standards of radiation protection practices [9, 10].

Managing CT radiation exposure is a crucial task, and both manual and automated approaches are available [14–16]. Manual techniques involve conducting retrospective surveys by extracting data from the picture archiving and communication system (PACS) or recording dose information manually if dose data not sent to the PACS. The gathered data are then analysed using basic statistical methods with software such as Microsoft Excel. However, manual processes are labour-intensive and susceptible to transcription errors [14–16]. To address these limitations, radiation dose monitoring software tools (DMS) have been developed. These automated tools, provided by various vendors, can either integrate directly with imaging equipment or interface with PACS, enabling the automatic collection and analysis of patient radiation dose data [14–18]. DMS tools provide features such as automatic dose benchmarking, calculating effective and organ-specific doses, tracking cumulative patient doses, generating alerts for dose threshold breaches, and producing detailed reports summarising dose levels across various imaging procedures [14–18]. Recent literature underscores the effectiveness of DMS in facilitating more efficient monitoring, evaluation, and continuous improvement of radiation safety practices [19–21]. International organisations such as the European Society of Radiology (ESR), advocate for the widespread adoption of DMS to enhance patient safety, strengthen quality assurance, and ensure regulatory compliance [22].

To the best of our knowledge, no studies have investigated CT dose data management practices or the prevalence of DMS tools in Australia. This study, therefore, aims to examine current CT dose management practices and the use of related tools, with the goal of providing valuable insights to inform policy development, promote best practices, and support dose management and optimisation initiatives.

## Materials and methods

This research obtained ethical approval from the University of Sydney’s Human Research Ethics Committee (Approval No. 2023/740). A cross-sectional, descriptive study design was utilised, employing an online survey to gather data. The survey instrument was based on a prior study conducted in Switzerland [23], with slight modifications to reflect the Australian healthcare environment. Before distribution, the survey was piloted with two healthcare professionals familiar with the target audience to ensure content appropriateness and to identify potential ambiguities. The survey included 20 items (19 multiple-choice and 1 free-text question) structured into four sections: [1] departmental demographics (e.g. number of CT scanners), [2] CT dose data collection and analysis practices, [3] use of DMS tools, and [4] radiation protection activities (Supplementary file S1). Inclusion criteria required respondents to be professionals directly involved in leading radiation protection activities, such as medical physicists, radiographers, radiation safety officers, or other qualified staff, within their radiology departments (public or private) operating at least one CT scanner. Screening questions at the beginning of the survey confirmed eligibility by assessing the respondent’s professional background, whether their department currently operates at least one CT scanner, and their involvement in radiation protection leadership or management.

According to the data published by the Australian Commission on Safety and Quality in Health Care (ACSQHC) on 31 December 2023, 32% (*n* = 1442 out of 4462) of accredited imaging facilities in Australia have CT scanners [24]. To reach to the leaders of radiation protection activities within these facilities, survey dissemination occurred through major Australian radiation safety organisations and networks including the Australian Radiation Protection and Nuclear Safety Agency (ARPANSA), Australian Society of Medical Imaging and Radiation Therapy, Australian College of Physical Scientists and Engineers in Medicine, the Agency for Clinical Innovation-Medical Imaging Network, and social media accounts of the Medical Image Optimisation and Perception Group, and all investigators. Recruitment materials included a flyer with a direct link and QR code leading to the participant information sheet, consent form, and survey. Participation was voluntary, with the right to withdraw at any time, and all data were collected anonymously from February to December 2024.

### Data cleaning and analysis

Following data collection, a data-cleaning process was conducted to include only complete and eligible responses. Incomplete responses, where participants voluntarily exited the survey early without providing sufficient data, were excluded. Responses from ineligible participants, whose surveys were automatically terminated because they were either not responsible for radiation protection activities, did not operate a CT scanner, or declined to participate, were also excluded. The remaining complete and eligible responses formed the final dataset, which was transferred to Microsoft Excel for descriptive analysis using frequency distributions and percentage calculations.

## Results

**Figure 1:**
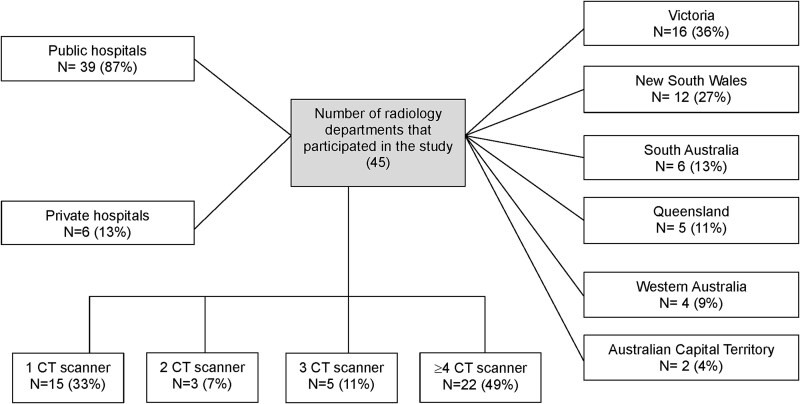
Overview of the type, location, and number of CT scanners in the radiology departments that participated.

Out of 132 responses received, 50 were excluded due to incompleteness, as participants voluntarily exited the survey without providing sufficient data for analysis. A further 37 responses were excluded due to ineligibility: not being responsible for radiation protection activities in their facilities (*n* = 33), not operating at least one CT scanner (*n* = 2), or declining to participate (*n* = 2). Ultimately, 45 eligible responses were analysed. Figure 1 summarises the distribution, types, and number of CT scanners across the participating radiology departments.

### Computed tomography dose data collection and analysis

**Figure 2:**
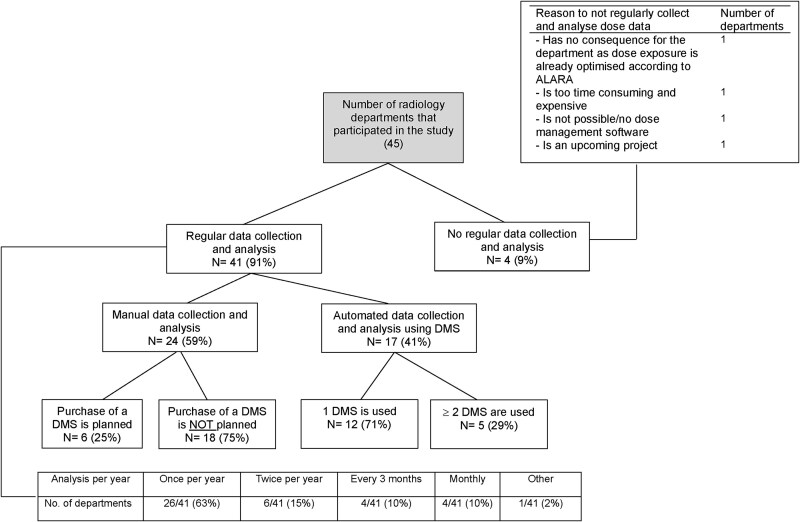
Methods for dose management and the frequency of dose assessment in participating radiology departments, along with the reported reasons for not conducting regular assessments.

Amongst the radiology departments that participated in the survey, 91% (*n* = 41/45) reported regularly assessing CT doses, while 9% (*n* = 4/45) did not, citing resource and time constraints (Fig. 2). Amongst those conducting regular assessments, the frequency varied, with 63% (*n* = 26/41) performing them annually. Additionally, 71% (*n* = 29/41) of these departments did not routinely include radiation exposure data in final CT reports. Only 12% (*n* = 5/41) reported consistently incorporating this information, and 17% (*n* = 7/41) included it upon request. When reported, the dose–length product (DLP) and CT Dose Index volume (CTDIvol) were the metrics most frequently mentioned, at 75% (*n* = 9/12) and 67% (*n* = 8/12), respectively. The effective dose was reported less often, at 50% (*n* = 6/12). Immediate dose evaluation after scanning was standard practice in only 27% (*n* = 11/41) of departments. Although 66% (*n* = 27/41) had the technical ability to assess doses immediately, they opted not to do so due to time and staffing limitations and 3% (*n* = 3/41) were unable due to system limitations.

### Utilisation of dose monitoring software

**Figure 3:**
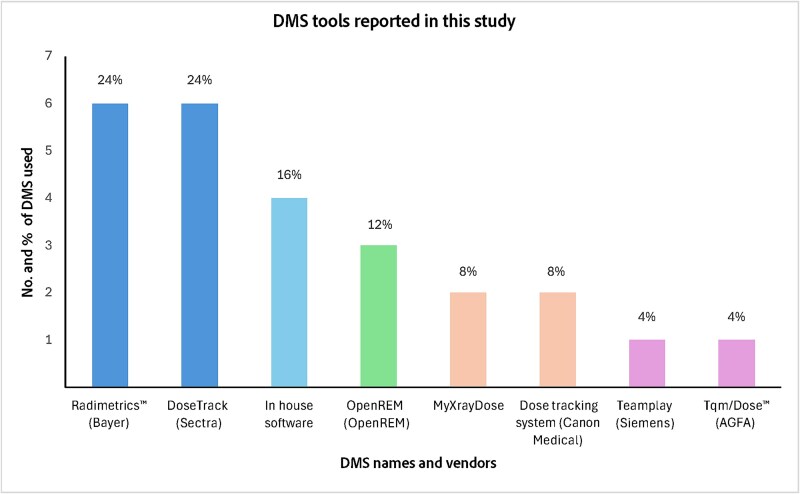
DMS systems used by the radiology departments that participated in the study, along with the number and percentage of each system used.

Amongst departments performing regular dose assessment, 41% (*n* = 17/41) utilised DMS tools for CT dose collection and analyses, 49% (*n* = 20/41) still relied on traditional data extraction from PACS and analysis through other software (e.g. Excel), and 10% (*n* = 4/41) used alternative methods. Only 25% (*n* = 6/24) of departments not using DMS reported plans to adopt such systems. Amongst the departments employing DMS, 71% (*n* = 12/17) utilised a single tool, while 29% (*n* = 5/17) operated multiple tools (three departments used two, one employed three, and another used four). Figure 3 illustrates the distribution of DMS tools utilised across the participating departments. Alongside CT dose assessment, departments also utilised DMS tools to evaluate radiation doses from other imaging modalities, including interventional radiology and fluoroscopy (76%, *n* = 13/17), X-ray (71%, *n* = 12/17), and mammography (47%, *n* = 8/17). Access to the DMS was highest amongst medical physicists (94%, *n* = 16/17), followed by radiographers (53%, *n* = 9/17) and IT specialists (35%, *n* = 6/17). In contrast, limited access was observed amongst chief physicians (29%, *n* = 5/17), consultants (18%, *n* = 3/17), and residents (12%, *n* = 2/17).

### Radiation protection activities

National DRLs from ARPANSA were utilised by 98% (*n* = 40/41) of departments conducting regular dose assessments. 49% (*n* = 20/41) had also developed local DRLs tailored to their equipment and protocols. A formal radiation protection team was established in only 41% of departments (*n* = 17/41). Radiographers (94%, *n* = 16/17) and medical physicists (82%, *n* = 14/17) were the most frequently included team members, while consultants (29%, *n* = 5/17), chief physicians (24%, *n* = 4/17), and residents (12%, *n* = 2/17) were less commonly involved. In nearly all cases (88%, *n* = 15/17), the dose team regularly communicated the department’s radiation exposure performance. Leadership of radiation protection activities was most often assigned to medical physicists (76%, *n* = 13/17), followed by radiographers (24%, *n* = 4/17).

## Discussion

Consistent radiation dose assessments is crucial for maintaining high standards of patient safety and ensuring compliance with radiation protection regulations [22]. The findings from the current study reveal that 91% (*n* = 41/45) of Australian radiology departments that completed the survey conduct regular CT dose assessments, reflecting a commitment to radiation safety and quality assurance. Nevertheless, the predominance of practices performing these dose reviews infrequently, typically once a year, suggests a need for more frequent dose assessment. Such practice facilitates the early detection of equipment problems, protocol issues, or operator mistakes, allowing timely actions and possible dose optimisation [18, 25]. Additionally, regular dose audits help identify excessive exposures that may need review and optimisation, as well as unexpectedly low doses that could require image quality assessment, thereby promoting a strong culture of safe radiation practice [26, 27].

A few respondents (9%, *n* = 4/45) admitted that regular dose monitoring and assessment was overwhelming due to cost, time, or lack of automation. The finding is unsurprising given that imaging facilities require additional support, improved funding, and more effective tools to remain compliant without overburdening their staff and clinical workflows. This finding highlights the need for institutional and governmental support to enable comprehensive dose management across all radiology settings. The use of automated dose monitoring systems is recommended to ensure consistent data collection and analysis, recognising the long-term efficiency gains and patient safety benefits of such tools [22]. Encouragingly, 41% (*n* = 17/41) of the departments surveyed had already adopted DMS systems, not only for CT but also for dose monitoring in modalities such as angiography, fluoroscopy, and mammography. The adoption of DMS tools offer significant advantages to dose review and management, including large-scale automated data collection and compliance tracking with adopted DRLs, which may enhance quality assurance. Whilst the immediate post-scan dose assessments offer ideal real-time feedback, these were infrequently performed across participating radiology departments due to workflow constraints. DMS can automatically detect examinations exceeding predefined dose thresholds and notify responsible staff, enabling prompt investigation of potential causes [16]. Alerts may arise from patient factors, such as large body habitus, orthopaedic hardware requiring higher doses for adequate image quality, or patient movement necessitating repeat scans [19, 28]. They may also result from operator factors, such as miscentering or scanning beyond the agreed region of interest, or radiologist factors, such as adding additional acquisitions [19, 28]. Each alert remains active until justified or verified within the DMS, typically through a user comment. This requirement to document the causes of high radiation events would enable radiology practices to identify areas that need improvement, such as protocol adjustments, improved staff training and education, which would strengthen quality assurance, and ultimately improve patient safety [19, 28]. A recent systematic review found that dose alerts from DMS helped many radiology practices quickly detect high CT radiation events and facilitated protocol amendments and other processes to enhance radiation protection [19]. These findings emphasise the need for Australian practices using DMS to set dose alert thresholds to support real-time dose monitoring and optimisation.

Amongst the participating departments, seven different DMS systems were in use as illustrated in Fig. 3; however, there are many other DMS tools from different vendors, which were not captured in the survey [14–18]. Despite differences in brand, most DMS platforms offer similar core functionalities, and the predominance of one company’s system over another is likely influenced by factors such as commercial marketing strategies or institutional preferences rather than substantial technical differences. Moreover, the widespread adoption of Radimetrics/Bayer and DoseTrack/Sectra systems can be attributed to these companies’ specialisation in imaging IT solutions and their development of software compatible with various CT scanner models and types. Meanwhile, the possible lower cost and customisation might explain why the in-house-built software was also commonly adopted amongst the participating departments. Regardless of the DMS vendor, initial and ongoing efforts are essential to ensure successful implementation and reliable operation. A recent systematic review on DMS use in CT dose management highlighted several recurring challenges faced by radiology departments that have adopted such systems. These challenges included data misclassification due to protocol nonstandardisation, invalid or missing data requiring checks and validation, compatibility issues necessitating manual corrections, and disconnections between the DMS and PACS/scanner, leading to challenges with data auto-updates [29]. These issues often required manual correction and routine validation to maintain analytical accuracy. Several studies have provided practical guidelines and recommendations to facilitate effective DMS implementation and minimise such issues [22, 29–31]. It is highly recommended to test the connection between CT equipment and the DMS, whether via PACS or direct integration, during installation, after software updates, and whenever new imaging systems are connected. This helps ensure that data are transmitted completely and accurately [22, 29–31]. Standardising CT protocol names across connected scanners is also crucial to enable accurate dose comparisons, reduce data misclassification, and support consistent analysis [22, 29–31]. Some DMS platforms offer name-mapping features that group different scan protocols under a single standard name, enabling meaningful patient dose audits even when multiple local protocol names for the same clinical indication are used. For systems lacking such features, resources like the RadLex Playbook or equivalent tools can help standardise CT protocol names. In addition, sufficient technical infrastructure, proper staff training, and multidisciplinary team cooperation are needed to maximise the utilisation of the DMS system and overcome potential challenges [29]. In this study, medical physicists and radiographers were the primary users of the DMS, which can be attributed to their direct involvement in dose monitoring and daily imaging operations. Expanding access to residents, referring physicians, and other clinicians could further strengthen radiation awareness across the team and maximise the benefits of such technology.

The inclusion of radiation dose metrics in radiology reports is regulated inconsistently worldwide; while some regions mandate their documentation, others provide no explicit requirement. For example, in the USA, California law has required since 2012 that CT dose metrics, including CTDIvol and DLP, be documented in both the patient’s radiology report and medical record [32, 33]. This mandate was introduced in response to several radiation overdose incidents and aims to prevent such events in the future [32, 33]. Similarly, the European Directive 2013/59/Euratom, effective from February 2018, requires that information on patient exposure be included in the medical radiological report and patient record, aiming to improve patients’ and professionals’ radiation awareness; yet, compliance across Europe remains inconsistent [9, 34]. In this study, most of the Australian imaging facilities surveyed (71%, *n* = 27/41) did not routinely include dose data in CT reports, which may be attributed not only to the absence of a national mandate in Australia but also to workflow barriers and limited perceived clinical utility. The value of recording and monitoring patients’ cumulative effective dose remains a topic of debate. The International Atomic Energy Agency (IAEA), together with other organisations, has issued a statement supporting the availability of relevant information from prior radiological procedures, including images, reports and dose history, particularly for patients undergoing repeated ionising imaging, to inform decision-making for new examinations [35]. The IAEA statement was based on recent evidence showing that a sizable number of patients receive concerningly high cumulative doses from recurrent imaging (e.g. ≥100 mSv) [6, 36, 37]. Other professional bodies, such as the American Association of Physicists in Medicine, the American College of Radiology and the Health Physics Society, generally align with the IAEA but argue that prior dose history should not be used as a determinant when justifying new imaging [38]. Instead, these professional bodies emphasise that imaging decisions must be guided by clinical need and prior imaging results, cautioning that reliance on radiation dose history could negatively impact patient care by deterring clinically beneficial imaging [38]. The UK Health Security Agency and the British Institute of Radiology share this position, except in rare cases where prior dose information may be relevant, such as multiple high-dose procedures (e.g. interventional or CT perfusion studies) performed on the same anatomical area within a short period, where deterministic effects may occur [39]. For example, a recent Australian case report of an 11-year-old with a ventricular assist device who underwent multiple brain CTs in 3 months (four noncontrast, two perfusion, and two angiographic), resulted in a lens dose of 516 mGy, exceeding the 500 mGy cataract threshold. Although all examinations were justified, the authors recommended implementing the DMS system to track patients’ doses, identify those at risk of exceeding tissue thresholds, support optimisation, and ensure appropriate follow-up [40]. While such individual cases highlight the potential risk of high cumulative doses and the potential benefits of dose tracking, international guidance offers a broader perspective. The International Commission on Radiological Protection (ICRP Publication 103) emphasised that medical exposures are not subject to dose limits and that effective dose is not intended for assessing individual patient risk [41]. Thus, cumulative dose history is best used to identify systematic issues such as repeated unjustified referrals, support dose optimisation, and contribute to research, rather than as a measure of individual patient risk. Adherence to radiation protection standards in clinical imaging practice is a shared responsibility that requires effective multidisciplinary cooperation. In this study, only 41% (*n* = 17/41) of imaging facilities reported having formal radiation protection teams, typically consisting of radiographers and medical physicists, with limited participation from physicians and residents. These findings emphasise the importance of broader adoption of such teams and stronger multidisciplinary collaboration to ensure consistent safety practices across Australian imaging facilities.

To the best of our knowledge, this is the first study to evaluate current practices (at the time of data collection, February to December 2024) regarding CT dose management and the adoption of DMS systems in Australia, offering valuable insights into the state of CT dose monitoring and management. The findings suggest that while most Australian radiology departments conduct regular CT dose assessments, demonstrating a strong commitment to radiation safety and quality assurance, these assessments are usually infrequent (often carried out only once a year) and depend largely on traditional methods. The most recent data from the Organisation for Economic Co-operation and Development show that, in 2021, Australia ranked amongst the developed countries with the highest access to CT scanners, at ~70 units per 1 million people [42]. This underscores the necessity for broader adoption of automated dose monitoring systems across radiological settings in Australia. Implementing such systems would facilitate more frequent and consistent dose assessments, enabling each facility to better evaluate their practices and identify areas needing improvement. Ensuring that imaging facilities have adequate support and funding will ease the dose assessment process and enhance their compliance with best practices in radiation protection. Lastly, establishing a dedicated radiation protection team in each imaging facility that uses ionising radiation is recommended, as this can strengthen teamwork, enhance radiation awareness, and promote safe imaging practices.

Several limitations should be considered when interpreting the findings of this study. Despite extensive promotion over 11 months through social media and various professional organisations, only 45 imaging facilities provided complete responses. Due to practical constraints, it was not feasible to contact all radiology departments directly; instead, the survey relied on third-party dissemination, which may have limited its overall reach. Additionally, participation was restricted to radiation protection leads in CT-using radiology departments. While this ensured input from individuals with relevant expertise, it also narrowed the target audience and may have contributed to the low response rate. The results indicate that DMS systems are not widely adopted across Australian radiology departments, possibly reflecting limited awareness or experience with these technologies, which may have contributed to uncertainty or a lack of engagement. These findings highlight the need for further research into awareness levels, perceived importance, and barriers to implementing DMS technologies in Australian clinical radiology settings.

## Conclusion

The findings demonstrate a commitment to best radiation safety practices in Australia through regular collection and analysis of CT dose data, but dose evaluations were often infrequent and relied on traditional methods. A broader implementation of automated dose monitoring systems, more frequent dose assessments and reporting, and the establishment of a dedicated radiation image optimisation team in each department may enhance radiation protection efforts and support continuous improvements in clinical imaging practice throughout Australia.

**Abbreviations:** 

CT	Computed tomography
DMS	Dose monitoring software
ESR	European Society of Radiology
IAEA	International Atomic Energy Agency
ALARP	As Low as Reasonably Practicable
DRLs	Diagnostic Reference Levels
PACS	Picture Archiving and Communication System
HREC	Human Research Ethics Committee
ACSQHC	Australian Commission on Safety and Quality in Health Care
ARPANSA	Australian Radiation Protection and Nuclear Safety Agency
ASMIRT	Australian Society of Medical Imaging and Radiation Therapy
ACPSEM	Australian College of Physical Scientists and Engineers in Medicine
ACI-MI	Agency for Clinical Innovation-Medical Imaging Network
MIOPeG	Medical Image Optimisation and Perception Group
PIS	Participant Information Sheet
DLP	Dose-Length Product
CTDIvol	CT Dose Index volume
AAPM	American Association of Physicists in Medicine
ACR	American College of Radiology
HPS	Health Physics Society
ICRP	International Commission on Radiological Protection
UKHSA	UK Health Security Agency
BIR	British Institute of Radiology
OECD	Organisation for Economic Co-operation and Development

## Supplementary Material

Supplementary_file_1_ncaf109

## Data Availability

The data underlying this article will be shared on reasonable request to the corresponding author.
