# Supplementary material for: Computed tomography dose management practices and the adoption of automated dose monitoring tools in Australia: a national survey
Source: Radiat Prot Dosimetry. 2025 Sep 23;201(17):1166–74. doi: 10.1093/rpd/ncaf109 (PMC12537306; doi:10.1093/rpd/ncaf109)
Supplement: Supplementary_file_1_ncaf109 [file supplementary_file_1_ncaf109.docx]

**Survey: Computed Tomography Dose Management Practices and the Adoption of Automated Dose Monitoring Tools in Australia: A National Survey**

**Primary Questions**

**I. Please state your professional background:**

- Medical Physicist

- Radiographer

- Other (please specify): _______

**II. Does your radiology department currently have at least one CT scanner?**

- Yes

- No (End of the survey)

**III. Does your role involve you leading the radiation protection activities in your radiology department?**

- Yes (will be allowed to participate in the survey)

- No (End of the survey)

**Section A: Part 1 - General Information on the Department**

**1. Which of these best describes your clinical centre?**

- Public hospital (in- and out-patient services)

- Private hospital (in- and/or out-patient services)

**2. In which Australian state/territory is your clinical centre located?**

- Australian Capital Territory

- New South Wales

- Northern Territory

- Queensland

- South Australia

- Tasmania

- Victoria

- Western Australia

**3.** **How many computed tomography (CT) scanners are currently in clinical use in your department (without PET/CT and SPECT/CT)?**

- 1

- 2

- 3

- 4

- Other (exact number) _______

**Part 2 - CT Dose Data Collection and Analysis**

*The following questions in this section aim to understand whether your radiology department has a regular practice regarding CT dose data collection/analysis or not, and how your department accomplishes this, whether manually or using dose-monitoring software (DMS) or other methods. Dose Monitoring Software (DMS) was developed by many manufacturers to monitor, collect, and report radiation dose information. DMS tools come in different names depending on the manufacturer such as DoseWatch (GE Healthcare), DoseWise (Philips Healthcare), Teamplay (Siemens), Radimetrics (Bayer), DoseTrack (Sectra), DoseMonitor (PACS Health), OpenREM (OpenREM), DoseM (Infinitt), DOSE (Qaelum), and others.*

**4. Does your department systematically collect and analyse CT radiation exposure data?**

- Yes (will be moved to the question N.6 and complete the survey)

- No (will be moved to the next question N.5 and then the survey will end)

**5.** **If you do not perform systematic collection and analysis, please state the reason:**

- Has no consequence for the department as dose exposure is already optimized according to ALARA.

- Is too time-consuming and expensive.

- Is not possible/no dose management software.

- Spot checks are performed occasionally.

- Is an upcoming project.

- Other (Specify): _______

**6.** **If you do perform systematic collection and analysis, how often are the assessments conducted? (Note: this question was kept open-ended to capture precise time intervals and the full variability in practices, as no prior data existed on current CT settings and practices, which may differ considerably between departments.)**

_____________

**7. Where do you collect the dose data of the CT scans?**

- In the PACS (Screenshot of displayed exposure data) and data transferred to an external database such as a dose monitoring software

- Only in the PACS (Screenshot of displayed exposure data)

- Different method (please specify): _______

**8. How do you analyse CT dose data?**

- Dose-Management-Software (DMS) (Will be allowed to complete part 3 use of Dose Management Software section)

- Manually (I do spot checks), but the purchase of a DMS is planned. (Will be Skipped to part 4 Radiation protection activities section)

- Manually (I do spot checks), and the purchase of a DMS is **not** planned yet. (Will be Skipped to part 4 Radiation protection activities section)

**Part 3 - Use of Dose Monitoring Software (DMS)**

**9.** **Which of the following vendors do you use their DMS in your clinical centre? (You can select more than one.)**

- DoseWatch™ (GE)

- DoseWise™ (Philips)

- Radimetrics™ (Bayer)

- Teamplay (Siemens)

- Dose tracking system (Canon Medical)

- Tqm/Dose™ (AGFA)

- DoseTrack (Sectra)

- DoseMonitor (PACS Health)

- OpenREM (OpenREM)

- DoseM (Infinitt)

- Other (Specify): ____________________________

**10.** **Who can access the DMS in the clinical routine?** (You can select more than one)

- Chief physician and deputies

- Consultants

- Residents

- Radiographers

- Medical physicists

- IT-specialists

- Data is part of a large external database, no direct access

**11.** **Which other imaging modalities do you analyse with a DMS? (**You can select more than one)

- Conventional X-ray

- Mammography

- Angiography/Interventional radiology

- Fluoroscopy

- DMS is only used for CT.

**Part 4 - Radiation Protection Activities**

**12.** **Do you have a dose team within your department? (a team that meets on a regular basis in order to improve radiation protection, e.g., specific dose data analysis to develop measures for further dose reduction)?**

- Yes

- No (will be Skipped to question 16)

**13.** **If you have a dose team, who are its members? (**You can select more than one)

- Chief physician and deputies

- Consultants

- Residents

- Radiographers

- Medical physicists

- IT-specialists

**14.** **If you have a dose team, who leads dose data analysis (either with a DMS or manually)?**

- Chief physician and deputies

- Consultants

- Residents

- Radiographers

- Medical physicists

- IT-specialists

**15.** **Do you communicate the results of dose data analysis within the department (e.g. by means of a meeting)?**

- Yes

- No

**16.** **Do you analyse dose data directly upon scan completion (i.e. do you assess whether an individual patient´s dose data lies within the Diagnostic Reference Levels)?**

- Yes

- No, but technically possible

- No, currently technically impossible

**17. Do you use Diagnostic Reference Levels (DRLs) provided by the Australian Radiation Protection and Nuclear Safety Agency (ARPANSA)?**

- Yes

- No

**18.** **Do you have your own local department-specific DRLs?**

- Yes

- No

**19. Do you add the patient's dosimetric information to the examination report?**

- Yes

- No

- Only when requested by the patient or referring doctor

**20.** **If yes, which parameters do you provide?**

- Total Dose-Length-Product (DLP)

- CT dose index (CTDIvol) of single series

- Size-specific dose estimate (SSDE)

- Effective dose (mSv)

- Other (Specify): ___________
